# Research Mapping of Trauma Experiences in Autism Spectrum Disorders: A Bibliometric Analysis

**DOI:** 10.3390/healthcare11091267

**Published:** 2023-04-28

**Authors:** Osvaldo Hernández-González, Andrés Fresno-Rodríguez, Rosario Elena Spencer-Contreras, Raúl Tárraga-Mínguez, Daniela González-Fernández, Francisca Sepúlveda-Opazo

**Affiliations:** 1 Faculty of Psychology and Institute of Humanistic Studies Juan Ignacio Molina, Universidad de Talca, Talca 3480094, Chile; 2 Faculty of Psychology, Universidad de Talca, Talca 3480094, Chile; afresno@utalca.cl (A.F.-R.); rspencer@utalca.cl (R.E.S.-C.); 3 Department of Education, School Management, Faculty of Teacher Training, University of Valencia, 46010 Valencia, Spain; 4 Faculty of Health Sciences, Universidad Autónoma de Chile, Santiago 7500912, Chile; dgonzalezf@uautonoma.cl; 5 Faculty of Health Sciences, Universidad de Talca, Talca 3480094, Chile; francisca.sepulveda@utalca.cl

**Keywords:** autism, bibliometrics, post-traumatic stress disorder, trauma

## Abstract

The number of research related to traumatic experiences in people with autism spectrum disorder (ASD) has grown exponentially, yet there are no bibliometric studies in this field. This article aimed to analyze the literature related to trauma and ASD published in Web of Science (WoS). Papers on trauma and ASD were retrieved from the WoS. Analysis and visualization of selected documents were performed using MS Excel (v16.0), VOS viewer (version 1.6.15), and R package (Biblioshiny, version 2.0). A total of 147 articles were included in this study. The results showed that production has been increasing over the last few years. Among the countries identified, the USA published the largest number of articles. Arvid Nikolai Kildahl, Sissel Berge Helverschou, and Liliana Dell’Osso were the authors with the most number of publications on this topic, and Autism was the most productive journal. The main research topics associated with ASD were post-traumatic stress and traumatic experiences in childhood. This bibliometric study contributes to understanding research trends on trauma and ASD by evaluating relevant publications in the last decades. The results of this bibliometric analysis can serve as a basis and orientation for new studies.

## 1. Introduction

Autism Spectrum Disorder (ASD) is characterized by difficulties in social interaction and verbal and nonverbal communication, restricted behaviors, and sensory problems [1]. The etiology of ASD is attributed to genetic, environmental, immunologic, perinatal, neuroanatomical, and biochemical factors [2]. Age and sex are variables that help to explain the variation in the constellation of symptoms that contribute to the broadening of the spectrum and, therefore, to the heterogeneity of the possible phenotypes consistent with the diagnostic criteria of ASD [3]. Epidemiological surveys worldwide show that the diagnosis of ASD has exponentially grown, with a proportion of one person out of 59 having this diagnosis [4]. People with ASD are more likely to suffer from psychiatric comorbidities and mental health problems [5,6]. The inherent characteristics of ASD may make affected individuals more vulnerable to traumatic experiences on multiple levels [7].

A trauma or traumatic event can be defined as exposure to actual or threatened death, serious injury, or sexual violence. This type of event may be experienced directly by the person or through witnessing the event occur to another person, learning that the traumatic event occurred to a close family member or close friend, or experiencing repeated exposure or extreme to aversive details of a traumatic event [1]. Such events include, but are not limited to, disasters, combat, serious accidents, torture, sexual violence, terrorism, assault or acute life-threatening illness; witnessing the threatened or actual injury or death of others in a sudden, unexpected, or violent manner; and learning about the sudden, unexpected or violent death of a loved one [8]. It is expected that experiencing a traumatic event generates a stress reaction; however, if the symptoms are maintained over time and alter the functioning of people, mental disorders can be diagnosed as post-traumatic stress disorder (PTSD) [9]. The possibility of experiencing a traumatic event during life is high (70.4%) [10]; however, the likelihood of developing PTSD depends on individual and contextual risk factors [11].

Some studies have highlighted that people with ASD are exposed to and end up being severely affected by traumatic events, especially abuse [12,13]. It has also been found that people with ASD are more vulnerable to PTSD and also have higher rates of PTSD than the general population [14,15,16]. There are several explanations for this, but the most compelling one is that pathognomonic cognitive and emotional features of ASD (for example, deficits in theory of mind, executive functioning, global processing, emotional understanding, and cognitive flexibility) may disturb the peritraumatic and post-traumatic processing, as well as assessment of traumatic memories, which increases the risk of developing PTSD [17]. Regarding the characteristics of ASD and the effects of trauma, it has been mentioned that traumatic experiences can exacerbate deficits related to communication, social interaction, and motor skills of daily living in children and youth with ASD [18]. A study by Storch et al. [19] highlighted that traumatic experiences are associated with suicidal thoughts and actions within the autistic population.

As for the diagnosis of PTSD, the findings of a systematic review conducted by Rumball [20] highlight that people with ASD can be diagnosed with PTSD according to current DSM-5 criteria, a finding that is corroborated in the systematic review by Kildahl [21]. In addition, Rumball’s review [20] provides preliminary evidence suggesting that traditional PTSD treatments may be effective with people with ADS.

Although the study of trauma and its consequences has been going on for several decades, its study in people with ASD is quite recent, and apparently, it is not fully systematized; to the best of our knowledge, no bibliometric analysis focused on traumatic experiences and ASD has been performed. The bibliometric analysis involves the mathematical and statistical analysis of the research literature [22] to highlight the most relevant articles, trends, countries, institutions, and authors with the greatest impact in any field of knowledge. Bibliometrics can complement previous literature reviews and thus provide us with a more global overview of traumatic experiences and ASD.

Considering the relevance of a bibliometric study to systematize recent research on trauma and ASD, this study aimed to analyze the scientific research published in WoS related to traumatic experiences and ASD. We envisage that results from the study will guide new researchers and experts in this field, as well as practitioners who work with people with ASD. The questions that guided this study were: Q1. What are the general characteristics of the literature on traumatic experiences and ADS? Q2. What is the trajectory of the annual publication? Q3. Who are the most productive authors in this field? Q4. What are the most productive journals? Q5. What were the most cited documents and the main results of each of them? Q6. What are the top keywords, co-occurrence network, and trending topics? Q7 Regarding the social structure, how are the collaboration networks of authors, institutions, and countries organized?

## 2. Materials and Methods

In this study, a bibliometric analysis of the literature published on the Web of Science (WoS) on the investigation of traumatic experiences in people with ASD was performed. Bibliometrics is a systematic, direct, and reproducible review method based on mathematics and statistics related to science, scientists, or scientific activity [23]. Due to its high indicative value for understanding the behavior of literature in a given field, the application of this technique in the field of trauma and ASD is growing every day [24].

### 2.1. Database Selection and Literature Search

WoS is a multidisciplinary and selective database that is made up of a variety of specialized indices. The main part of the WoS platform is the Core Collection (WoS CC). Due to its intellectual value, we selected it to analyze the publications related to traumatic experiences and ASD. The search used the advanced search interface: “Article Title, Abstract, Keywords”. Search terms were strategically combined using Boolean operators: “autism spectrum disorder” OR “autism” AND “trauma” OR “post-traumatic stress disorder” OR “traumatic experiences”. The search in WoS included the period (1975–2022). The search was performed by two of the main authors on 13 September 2022. A total of 448 articles potentially relevant to the study were retrieved.

### 2.2. Inclusion/Exclusion Criteria

Articles studying traumatic experiences in samples of children, adolescents, and adults with ASD, and close people, such as family members and caregivers, were included. No language or publication time restrictions were applied. Articles dealing with traumatic experiences in people with other neurodevelopmental disorders were excluded. Also, we excluded papers that discussed problem behaviors but were not necessarily linked to traumatic experiences.

### 2.3. Preliminary Search Evaluation

**Figure 1:**
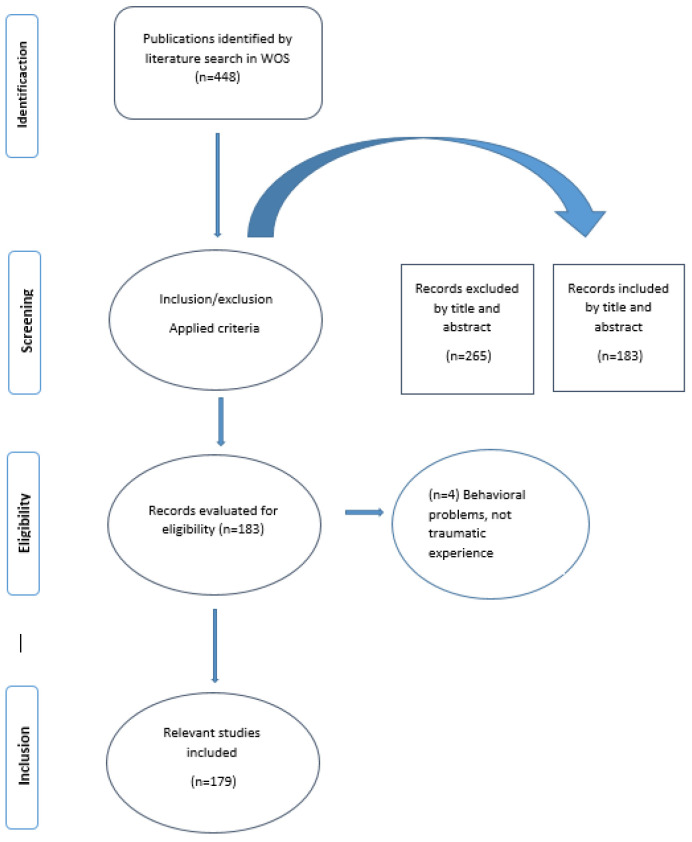
Flowcharts of four phases of the publication data extraction and filtering process.

In order to reduce selection bias, the 448 articles collected in the initial search were independently evaluated by two authors. Eligibility and relevance were evaluated for each record, which allowed the exclusion of 301 documents. The double review resulted in 94% agreement. Articles where ratings differed were discussed, and agreement was reached between the two manuscript reviewers. Once the process was completed, 179 records were selected for the bibliometric analysis (see selection path in Figure 1).

### 2.4. Data Preparation and Analysis

SPSS 21.0 software was used to analyze the trend of publications by year. In addition, MS Excel (v16.0) and R packages (Biblioshiny, version 2.0) were used. Data were exported to different file formats for analysis (e.g., BixTex, CSV). Analysis indicators included publication number, average citation per publication, countries, institutions, journals, keywords, authors, and main documents. Lokta’s law was applied to see the productivity of authors. According to Ahmad et al. [25], Lotka’s Law shows that an uneven distribution exists so long as most articles are focused on a small portion of highly productive authors. Bradford’s law was applied to describe the main journals. Bradford’s Law (1934) establishes the dispersion of the scientific literature since it orders the scientific journals in decreasing order of productivity of articles in a given field of study. Various measurement indicators are also used, such as the h-index (an author-level metric that attempts to measure both the productivity and citation impact of a scientist’s or scholar’s publications), the g-index (quantifies bibliometric productivity based on the authors’ publication history), m-index (this value represents the average amount that the author’s h-index has increased per year over the author’s publishing career and can help differentiate between two authors with similar h-indexes but different career lengths), TC (Total citations, the growth rate shows the productive increase, it is the percentage difference in the number of papers relative to the previous period), NP (Number of publications), and PY (Start of publication year). We also use the co-occurrence network (clustered interconnection of terms) and the thematic map (diagrams representing words, ideas, tasks, or other concepts linked and arranged radially around a keyword or central idea) to understand research trends in the subject matter.

### 2.5. Ethical Statement

This study did not involve the participation of human beings and did not require the approval of an ethics committee to carry it out.

## 3. Results

### 3.1. Output of General Information and Annual Publication

**Figure 2:**
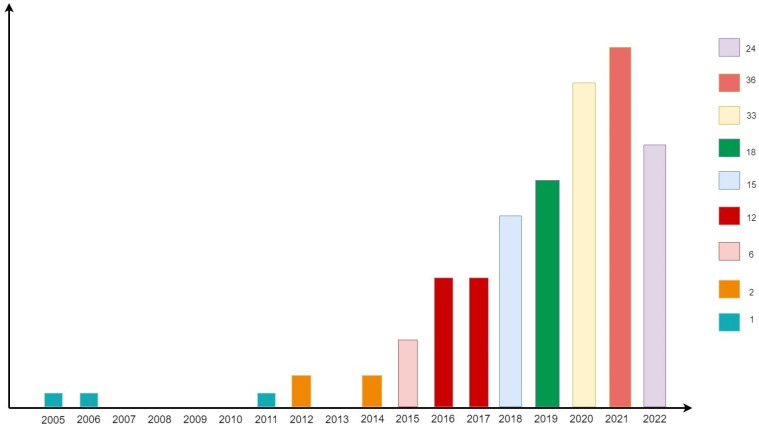
Number of annual publications related to trauma and ASD research from 1976 to 2022.

After screening, a total of 147 records were identified from the WoSCC Science Citation Index Expanded database. The main information of the sample was distributed as follows: MAIN INFORMATION ABOUT DATA (Timespan 2005:2022, Sources (Journals) 95, Documents 179, Document Average Age 3.88, Average citations per doc 9.279, References 1); DOCUMENT CONTENTS (Keywords Plus (ID) 419, Author’s Keywords (DE) 369); AUTHORS (Authors 468, Authors of single-authored docs 29); AUTHORS COLLABORATION (Single-authored docs32, Co-Authors per doc 3.78, International co-authorships% 13.41); DOCUMENT TYPES (articles 108, book chapter 1, articles early access 15, proceedings paper 2, book review 1, editorial material 5, letter 1, letter early access1, meeting abstract 19, news item 1, review 24, review early access 1). The number of annuals has grown in the last decade. Figure 2 shows that WoS posts on traumatic experiences in ASD appeared in 2005. During the last 5 years (2016–2021), there has been a sustained increase in publications on the subject, with 2021 being the most productive year. Although 2022 is not complete, it was selected to see and compare the publication rate with previous years. The annual growth rate is 20.56%.

### 3.2. Distribution of Authors

In total, 468 authors were responsible for the analyzed publications. Table 1 shows the authors with the highest number of publications. The most productive authors were Arvid Nikolai Kildahl, Sissel Berge Helverschou, and Liliana Dell’Osso, with more than five articles published. The h-index and the total number of citations considered a measure of influence and performance [26] support that these authors have been the most productive on this topic. However, the most internationally cited authors were Connor M. Kerns, Steven J. Berkowitz, and Arvid N. Kildahl.

**Figure 3:**
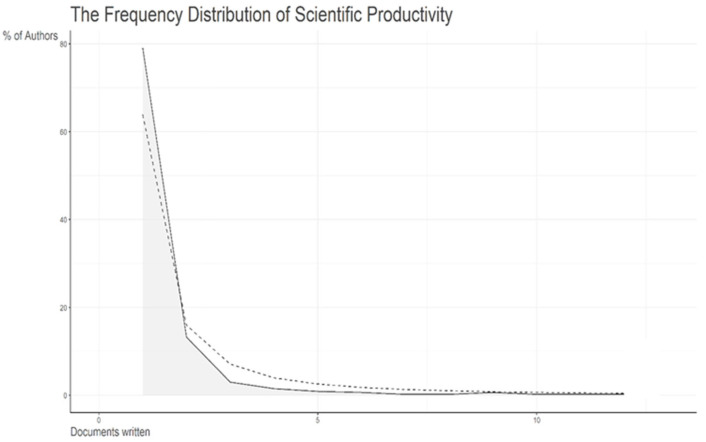
Lotka’s law.

In Figure 3, it is possible to observe the approximation between the observed and expected values of the distribution of author productivity. It is visible that there is a correspondence between the observed and expected scientific production, so we can conclude that Lokta’s law is fulfilled.

### 3.3. Journal Distribution

In the analyzed data, 95 journals were involved with the publication of documents on traumatic experiences and ASD in WoS. Table 2 shows that the journal Autism published the largest number of documents (11), followed by the Journal of Autism and Developmental Disorders (10) and Research in Developmental Disabilities (9). We also use Bradford’s law to describe top journals. Bradford’s law is related to the dispersion of scientific literature. For example, if scientific journals are arranged in decreasing order of productivity of articles in a given field of study, they can be divided into a core of journals dedicated more particularly to the subject, where those in zone one are the most prominent journals [27].

**Figure 4:**
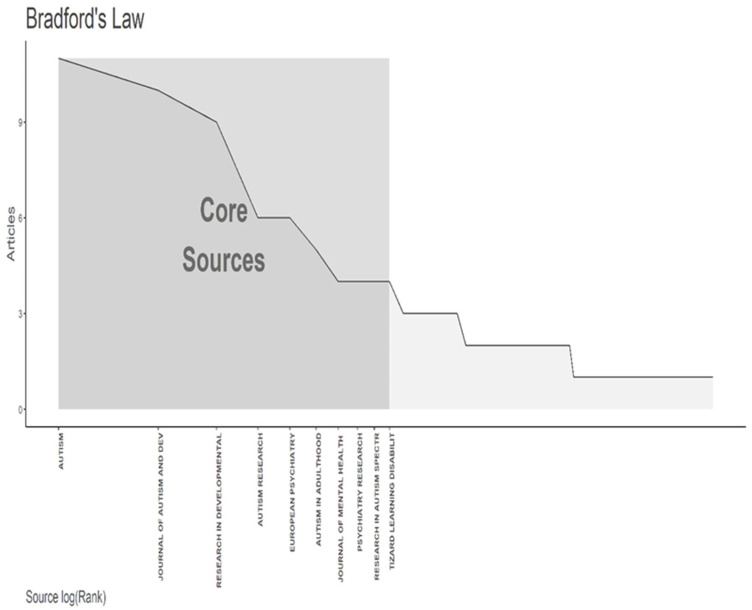
The most prominent grassroots journals in trauma and ASD research are based on Bradford’s Law.

Our analysis reveals that core one encompasses 10 journals, among which Autism, Journal of Autism and Developmental Disorders, and Research in Developmental Disabilities are the most prominent (see Figure 4).

### 3.4. Most Cited Documents Worldwide

Table 3 shows the most cited documents in the field. The most cited manuscript (119 citations) was by Connor Morrow Kerns, Craig J. Newschaffer & Steven J. Berkowitz [17]. This review article proposes a conceptual framework for understanding the interaction between ASD, trauma, and traumatic sequelae and presents recommendations for future research. The second most cited study (60 citations) was written by Mohamad Mehtar & Nahit Motavalli [28]. In this study, it was found that witnessing or being a victim of accidents/disasters/violence was the most common type of trauma. Interestingly, the rate of sexual and/or physical abuse was less than in the general population. Trauma history and PTSD rates were higher in girls than boys. Deterioration in social and communicative abilities, increase in stereotypes, aggression, distractibility, sleep disorders, agitation, hyperactivity, self-injury, and loss of self-care skills were the most common symptoms detected following trauma. The article that received the most citations in the least amount of time (effectiveness) was the study carried out by Freya Rumball, Francesca Happé & Nick Grey [7]. The results of this study indicated that trauma-exposed ASD adults are more at risk of developing PTSD compared to the general population. More than 40% of ASD individuals were found to have PTSD symptom scores above the cutoff for possible PTSD, following DSM-5 or non-DSM-5 trauma experiences.

### 3.5. Conceptual Structure: Co-Occurrence Network Analysis and Thematic Map

**Figure 5:**
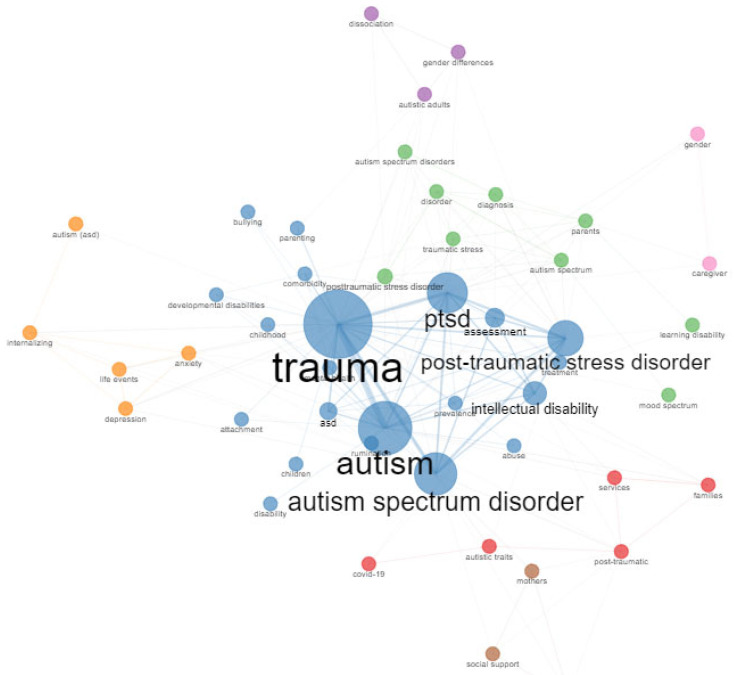
Keyword Co-Occurrence Network (KCN).

The Keyword Co-occurrence Network (KCN) provides insight into thematic trends in the field of trauma and ASD. It allows an understanding of the components and structure of a scientific field by examining the links between keywords in the literature, as well as the strength of their connections [34]. In Figure 5 it can be seen that the word “trauma” in first place and “PTSD” in second place are the words most used by authors to help indexers and search engines to find relevant articles in the field of autism. In addition, a wide range of topics, such as mental health, assessment, diagnosis, parenting, and treatment, among others, can be associated with these search engines.

**Figure 6:**
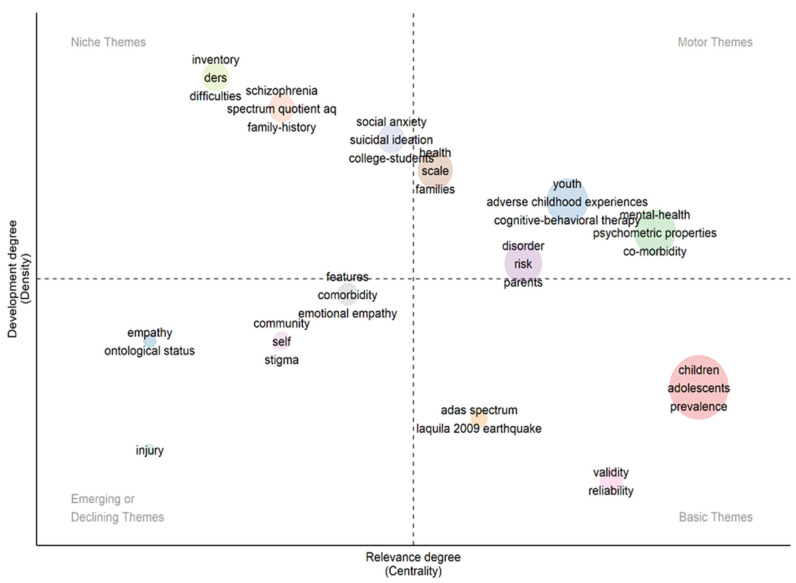
Thematic map.

The thematic map of the data was analyzed in order to obtain an overview of research trends and their sustainability over time. Centrality underlines the importance of a topic in the field of study, and density indicates the development of the topics. The volume of each sphere reflects the number of articles using this keyword. Figure 6 shows four quadrants that reflect different levels of centrality and density. The motor themes are those that are well developed and, therefore, central to the structuring of trauma and autism research. In this quadrant, we identified that mental health, adverse childhood experiences, cognitive behavioral therapy, and psychometric properties have been deeply studied. The basic themes are represented by central and undeveloped themes. In this quadrant, we can see that, as a striking result, there is a need to further develop the prevalence of trauma in the childhood and adolescence of autistic people. For example, empathy and communitarianism seem to be emerging themes in this field of study. On the other hand, specialized themes are related to deficits, family history, suicidal ideation, social anxiety, and school settings.

### 3.6. Social Structure: Collaboration Network of Authors, Institutions, and Countries

**Figure 7:**
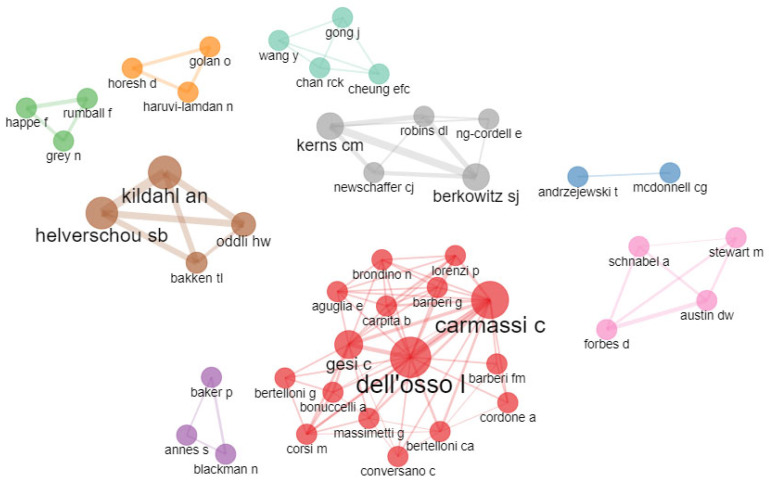
Collaboration network of the most representative authors in research related to trauma and ASD.

The main collaborative networks are composed of a total of 44 authors. It is possible to observe that the main collaborative network is represented by the red cluster, in which Liliana Dell’Osso, Claudia Carmassi, and Camilla Gesia stand out. The networks represented by the light green cluster composed mainly by Connor Morrow Kerns and Steven J. Berkowitz, and the light pink cluster composed mainly by Arvid Nikolai Kildahl and Sissel Berge follow in importance (see Figure 7).

**Figure 8:**
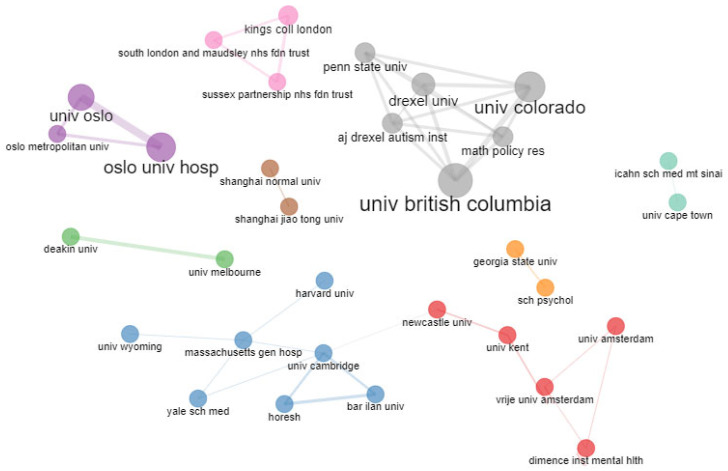
Collaboration network of the most represented institutions that carry out research in trauma and ASD.

There are 35 institutions participating in ASD and trauma-related scientific production registered with WoS. Figure 8 shows that the main institutional collaboration networks are mainly grouped into four clusters. The most representative institution of the purple cluster is The University of British Columbia, the orange cluster Oslo University Hospital—Oslo Universitetssykehus, the gray cluster King’s College London, and the blue cluster University of Cambridge.

**Figure 9:**
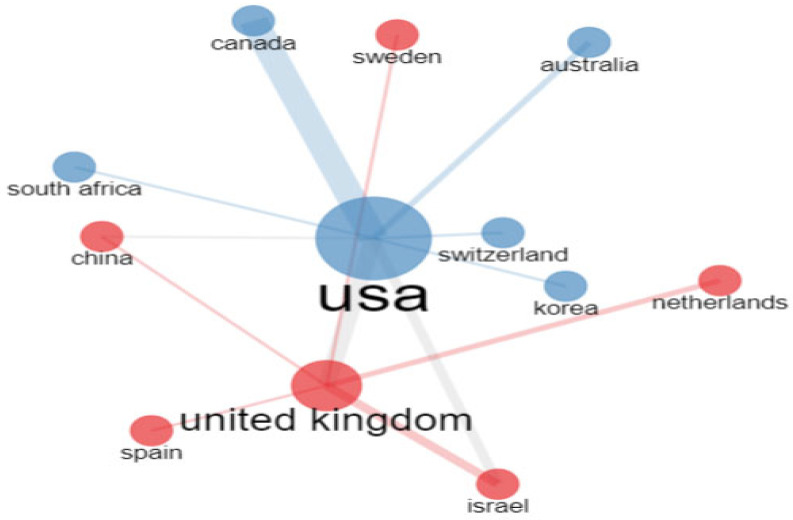
Collaboration network of the most representative countries in research related to trauma and ASD.

The United States is the country with the highest number of collaborations, followed by the United Kingdom. The countries that have participated in these studies are mainly European and North American. Two Asian countries (Korea and China), Australia, and Israel are also represented (see Figure 9).

## 4. Discussion

The objective of this biometric article was to analyze the literature related to trauma and ASD published in WoS from 1976 to 2022. Our results suggested that the original articles were the main means of scientific dissemination. The fact that the results are presented through this means of scientific dissemination indicates that the research is well established with an adequate theoretical and research base. Also, the number of publications showed a growth trend from 2015, since before that date, it had been a neglected topic in the WoS base. This trend has been verified in other bibliometric analyzes related to ASD [35,36]. The results of 2022 suggest that this field of research may continue to be a topic of great interest for researchers in the coming years.

Based on the analysis of the authors, we found that those with more publications do not necessarily have higher centrality and citation frequency (with the exception of Arvid Nikolai Kildahl), which indicates that the academic influence of the authors may depend on many variables. Regarding the journals that have been published on this topic, the distribution of literature was concentrated in the first 10 journals. These journals can be divided into three categories: ASD journals, disability journals in a general sense, and psychiatry journals. This demonstrates a growing interest in the subject that captures the attention of journals with different scopes, which could imply a favorable projection for this field of study with emphasis on the mentioned topics. However, it should be noted that due to the novelty of the subject, there is not a large volume of articles published in journals on trauma and ASD, but growth has been observed in the last five years. This indicates that in the coming years, these journals could show a growing interest in receiving these types of articles. These journals have also had an important place in other bibliometrics dedicated to ASD [37,38].

The most cited studies were published between 2011 and 2020. The topics were related to traumatic events in childhood and the prevalence rates of post-traumatic stress related to the victim of an accident, disaster, violence, and/or abuse. According to the criteria of Milojević [39], the age of the articles is consistently related to the number of citations received. Although these articles, considering the publication timeline, were published in the initial period of growth and therefore partially comply with this characteristic. The originality of its contents must be highlighted since, in 2022, these same topics will continue to be a trend [40,41]. This means that these articles constitute an important theoretical basis for recent studies. To some extent, the validity and interest of the scientific community in this topic could explain why these documents have been highly cited and would not only be explained by the time variable. Therefore, more research is still needed on these issues from an evaluation and intervention point of view. The results of the keyword co-occurrence analysis revealed that the research on the subject selected as the object of study is carried out in two groups: (1) trauma (understanding trauma as a general category that accounts for events or experiences considered traumatic during childhood or adulthood) and ASD, (2) post-traumatic stress (mainly PTSD symptoms) and ASD. These two groups demonstrate the main aspects of the research field.

The analysis of the thematic map allowed us to identify which are the driving, specialized, basic, and emerging themes related to the study of trauma and autism. Results evidenced that the study of empathy is an emerging line of research in the field of trauma and autism. These findings are consistent with what was stated by Hume & Burgess [42], who propose that future studies address the possible interactions between autism, trauma, PTSD, and affective empathy, to understand whether autistic adults with PTSD can recover affective empathy after treatment interventions.

The most productive institutions are located in various countries, such as Norway, the USA, the UK, and Italy. A majority participation of culturally Western countries was observed, with the exception of China and Israel. This suggests that there is room for partnerships to be established with researchers from developing countries that represent cultures other than those mentioned to advance the study of trauma and ASD. Collaborations like these could reveal the specific characteristics of traumatic experiences and their effects on ASD people in contexts other than those studied so far [43].

This study had some limitations that must be considered when interpreting the results. First, only publications from the WoS database were used as units of analysis, which could lead to incomplete literature searches. For future studies, it would be key to use other international databases, such as PubMed. Finally, although the document selection process was carried out by two authors independently and in parallel, a certain bias in the selection of publications cannot be ruled out.

## 5. Conclusions

The present study highlighted the state of research published in WoS related to traumatic experiences and ASD, as well as current research hotspots and trends. Traumatic experiences in people with ASD is a relatively new field, but the evidence indicates that this line of research will continue to be updated, driven by the need to understand the explanatory bases of the life experience of people with ASD. The research has been developed mainly in Western countries. Traumatic experiences in childhood and post-traumatic stress disorder have consolidated as historical lines of research. The research trends analysis indicated that the study of empathy and its relationship with trauma and PTSD is an emerging line of research relevant to consider in the field of autism. Due to its bibliometric nature, this study can serve as a basis to guide new researchers to find possible collaborators and also guidance to design studies that provide valuable information for researchers and professionals working in the field of ASD.

## Figures and Tables

**Table 1:** Most prolific authors.

Authors	Articles	Fractionalized Articles	h_Index	g_Index	m_Index	TC	NP	PY_Start	Institution
Kildahl AN	12	3.50	7	10	1.400	136	10	2019	Oslo University Hospital
Helverschou SB	11	3.00	7	10	1.400	136	10	2019	Oslo University Hospital
Dell’osso L	10	1.28	6	9	0.857	108	9	2017	University of Pisa
Berkowitz SJ	9	1.87	3	5	0.429	256	5	2015	University of Pennsylvania
Carmassi C	9	1.14	4	8	0.571	77	8	2017	University of Pisa
Kerns CM	9	1.87	3	5	0.427	256	5	2015	University of British Columbia
Oddli HW	8	2.33	5	7	1.250	77	7	2020	University of Oslo
Bakken TL	7	1.67	6	7	1.200	121	7	2019	Oslo University Hospital
Gesi C	6	0.71	4	6	0.571	92	6	2017	University of Pisa
Robins DL	6	1.06	1	1	0.333	2	2	2021	Drexel Autism Institute

Note: TC (Total citations, the growth rate shows the productive increase, it is the percentage difference of the number of jobs in relation to the previous period); NP (Number of publications); PY (Publication year start).

**Table 2:** Top 10 journals that have published articles on trauma and ASD.

Sources	Articles	h_Index	g_Index	m_Index	TC	NP	PY_Start	Country	Scimago Quartile
Autism	11	6	10	1.345	153	10	2010	UK	1
Journal of Autism and Developmental Disorders	10	8	9	0.889	336	9	2015	USA	1
Research in Developmental Disabilities	9	4	6	1.000	47	9	2020	USA	2
Autism Research	6	5	5	1.250	132	5	2020	USA	1
European Psychiatry	6	2	2	0.286	8	4	2017	UK	1
Autism in Adulthood	5	2	3	0.667	13	3	2021	USA	2
Journal of Mental Health Research in Intellectual Disabilities	4	4	4	0.800	78	4	2019	UK	2
Psychiatry Research	4	4	4	0.571	78	4	2017	IE	1
Research in Autism Spectrum Disorders	4	2	2	0.154	68	2	2011	NL	1
Advances in Autism	4	3	3	0.500	50	3	2018	UK	3

Note: h-index (author-level metric that attempts to measure both the productivity and citation impact of the publications of a scientist or scholar; g-index (quantifies the bibliometric productivity based on the publication history of authors); m-index (this value represents the average amount the author’s h-index has increased per year over his or her publishing career and can help differentiate between two authors with similar h-indexes but different career lengths); TC (Total citations, the growth rate shows the productive increase, it is the percentage difference of the number of jobs in relation to the previous period); NP (Number of publications); PY (Publication year start).

**Table 3:** The characteristics and main results of highly cited papers on research related to trauma and ASD.

Authors (Year)	Journal	Title	Total Citations	TC per Year	Normalized TC	Principal Result
Kerns, C.M., Newschaffer, C.J., & Berkowitz, S.J. (2015) [17]	Journal of Autism and Developmental Disorders	Traumatic Childhood Events and Autism Spectrum Disorder	119	13.22	1.92	Traumatic childhood events were associated with a wide range of negative physical, psychological, and adaptive outcomes over the life course and were one of the few identifiable causes of psychiatric illness.
Mehtar, M., & Mukaddes, N.M. (2011) [28]	Research in Autism Spectrum Disorders	Post-traumatic Stress Disorder in individuals with a diagnosis of Autistic Spectrum Disorders	60	4.62	1.00	Witnessing or being a victim of accidents/disasters/violence was the most common type of trauma. Interestingly, the rate of sexual and/or physical abuse was less than in the general population. Trauma history and PTSD rates were higher in girls than boys. Deterioration in social and communicative abilities, increase in stereotypes, aggression, distractibility, sleep disorders, agitation, hyperactivity, self-injury, and loss of self-care skills were the most common symptoms detected following trauma.
Roberts, A.L., Koenen, K.C., Lyall, K., Robinson, E.B., & Weisskopf, M.G. (2015) [29]	Child Abuse & Neglect	Association of autistic traits in adulthood with childhood abuse, interpersonal victimization, and post-traumatic stress	55	6.11	0.89	Compared to the general population, a sample of women with autistic traits were more likely to have been sexually abused (40.1% versus 26.7%), physically/emotionally abused (23.9% versus 14.3%), mugged (17.1% versus 10.1%), pressured into sexual contact (25.4% versus 15.6%) and have high PTSD symptoms (10.7% versus 4.5%). Levels of autistic traits are associated with abuse, trauma, and PTSD symptoms.
Taylor, J.L., & Gotham, K.O. (2016) [13]	Journal of Neurodevelopmental Disorders	Cumulative life events, traumatic experiences, and psychiatric symptomatology in transition-aged youth with autism spectrum disorder	49	6.13	3.75	Over 50% of youth had experienced at least one trauma. Nearly one-half had clinical-level mood or anxiety symptomatology. There was a statistically significant relation between the absence/presence of trauma and mood symptomatology; nearly 90% of the youth with clinical-level mood symptoms had at least one trauma, compared to 40% of those with no mood symptomatology.
Hoover, D.W., & Kaufman, J. (2017) [30]	Current Opinion	Adverse childhood experiences in children with autism spectrum disorder	47	7.83	2.99	Children with ASD with an elevated number of ACES also experience a delay in ASD diagnosis and treatment initiation. There is no evidence of an increased risk of child maltreatment within the ASD population.
Rumball, F., Happé, F., & Grey, N. (2020) [7]	Autism Research	Experience of Trauma and PTSD Symptoms in Autistic Adults: Risk of PTSD Development Following DSM-5 and Non-DSM-5 Traumatic Life Events	40	10.00	3.29	Thirty-three people reported experiencing a “DSM-5” traumatic event (i.e., an event that met DSM-5 PTSD Criterion A), and 35 reported a “non-DSM5” traumatic event. Trauma-exposed adults with ASD were found to be at increased risk of developing PTSD, compared with previous statistics from the general population, with PTSD symptom scores crossing thresholds suggesting a probable PTSD diagnosis for more than 40% of people with ASD who follow the DSM-5 or not DSM-5 trauma.
Hoover, D.W. (2015) [31]	Review Journal of Autism and Developmental Disorders	The Effects of Psychological Trauma on Children with Autism Spectrum Disorders: A Research Review	38	4.22	0.61	Bullying has received much attention, while there is a paucity of research on other types of trauma. Anxiety, social isolation, and developmental regression are associated with trauma.
Kupferstein, H. (2018) [32]	Advances in Autism	Evidence of increased PTSD symptoms in autistics exposed to applied behavior analysis	34	5.67	2.16	This study noted PTSS in nearly half of ABA-exposed participants, while non-exposed controls had a 72 percent chance of being asymptomatic. ABA satisfaction ratings for caregivers averaged neutral or mild satisfaction. In contrast, adult satisfaction with ABA was lower on average and also tended to take on either extremely low or extremely high ratings.
Zhang, W., Yan, T.T., Barriball, K.L., While, A.E., & Liu, X.H. (2013) [33]	Autism	Post-traumatic growth in mothers of children with autism: A phenomenological study	34	3.78	0.55	A new philosophy of life, appreciation of life, relating to others, personal strength, and spiritual change were five domains of post-traumatic growth in mothers of children with autism. Perceived social support, peer example, effective coping style and self-efficacy enhancement were facilitating factors of post-traumatic growth.
Kildahl, A.N., Bakken, T.L., Iversen, T.E., & Helverschou, S.B. (2019) [21]	Journal of Mental Health Research in Intellectual Disabilities	Identification of Post-Traumatic Stress Disorder in Individuals with Autism Spectrum Disorder and Intellectual Disability	29	5.80	2.03	The assessment methodology in the studies varied, as did the symptom reporting format. The assessment methodology in the studies varied, as did the symptom reporting format. The DSM-5 criteria provide a useful framework for integrating study results, indicating that PTSD can be identified in people with ASD and ID. However, symptoms involving disturbances in arousal and negative disturbances in thought and behavior appear to be more readily identifiable than re-experiencing and avoidance symptoms.

## Data Availability

The data presented in this study are openly available in Zenodo at https://zenodo.org/record/7660174#.ZEtoVHZBy70 (accessed on 21 February 2023).
